# Exome Sequencing in Fetuses with Structural Malformations

**DOI:** 10.3390/jcm3030747

**Published:** 2014-07-08

**Authors:** Fiona L. Mackie, Keren J. Carss, Sarah C. Hillman, Matthew E. Hurles, Mark D. Kilby

**Affiliations:** 1 Centre of Women’s and Children’s Health & School of Clinical and Experimental Medicine, College of Medicine and Dentistry, University of Birmingham, Edgbaston, Birmingham B15 2TT, UK; E-Mails: sarahchillman@hotmail.com (S.C.H.); m.d.kilby@bham.ac.uk (M.D.K.); 2 Fetal Medicine Centre, Birmingham Women’s Foundation Trust, Edgbaston, Birmingham B15 2TT, UK; 3 Genome Mutation and Genetic Disease Group, The Wellcome Trust Sanger Institute, Wellcome Trust Genome Campus, Hinxton, Cambridge CB10 1SA, UK; E-Mails: kc7@sanger.ac.uk (K.J.C.); meh@sanger.ac.uk (M.E.H.)

**Keywords:** exome sequencing, prenatal, fetus, prenatal diagnosis

## Abstract

Prenatal diagnostic testing is a rapidly advancing field. An accurate diagnosis of structural anomalies and additional abnormalities in fetuses with structural anomalies is important to allow “triage” and designation of prognosis. This will allow parents to make an informed decision relating to the pregnancy. This review outlines the current tests used in prenatal diagnosis, focusing particularly on “new technologies” such as exome sequencing. We demonstrate the utility of exome sequencing above that of conventional karyotyping and Chromosomal Microarray (CMA) alone by outlining a recent proof of concept study investigating 30 parent-fetus trios where the fetus is known to have a structural anomaly. This may allow the identification of pathological gene anomalies and consequently improved prognostic profiling, as well as excluding anomalies and distinguishing between *de novo* and inherited mutations, in order to estimate the recurrence risk in future pregnancies. The potential ethical dilemmas surrounding exome sequencing are also considered, and the future of prenatal genetic diagnosis is discussed.

## 1. Introduction

The incidence of congenital abnormalities in the UK is approximately 2.2% [1]. These are frequently first identified by ultrasound scan during pregnancy. There is a wide range of potential outcomes for fetuses with abnormalities. Some abnormalities, such as isolated cleft lip, can be corrected in early childhood with a surgical procedure, and often has minimal long-term impact on the child [2]. Other abnormalities, such as bilateral renal dysplasia or cerebral malformations, are associated with high morbidity and mortality [3,4]. There is little doubt that a chromosomal anomaly associated with a structural malformation significantly worsens prognosis.

Numerous genetic mutations have been associated with fetal structural abnormalities. These include aneuploidies, copy number variants (CNVs), loss-of-function single nucleotide variants (SNVs) and mis-sense SNVs [5,6,7,8,9]. Knowing the cause of a fetal structural abnormality may help clinicians to make an accurate prognosis regarding the pregnancy, and estimate recurrence risk for any future pregnancies. This helps the families to make informed decisions, including whether to terminate the pregnancy. Despite the importance of a diagnosis, currently only a minority of children affected by a developmental disease receive a genetic diagnosis, to the frustration of families, clinicians and researchers alike [10].

In this review, we will outline the current approaches used to investigate the genetic cause of prenatal structural abnormalities. We will then describe several studies that have recently used next-generation sequencing (NGS) for this purpose, and we will discuss advantages and disadvantages of this approach. Finally, we will outline some ethical issues that this technology raises, and discuss likely future directions of the prenatal genetic diagnostics field.

## 2. Current Techniques for Prenatal Genetic Diagnosis

### 2.1. Sampling Methods

Fetal DNA for genetic testing may be obtained invasively or non-invasively. Invasive methods include amniocentesis, fetal blood sampling and chorionic villus sampling (CVS). They yield cells from which fetal genomic DNA can be extracted. The major disadvantage of invasive sampling is that the risk of miscarriage increases by around 1% following a procedure [11,12,13,14]. Alternatively, fragmented cell-free fetal DNA (cffDNA) can be obtained non-invasively from maternal plasma [15], but there are presently limitations to its use in prenatal diagnostics.

### 2.2. Karyotyping

One invaluable tool for the detection of chromosomal aberrations associated with fetal and congenital abnormalities is karyotyping, where whole chromosomes are stained and examined using a light microscope. In classical cytogenetics, the stains (such as Giemsa (G-) stain) reveal patterns of light and dark bands that are unique to each chromosome. The technique was developed in the late 1960s and early 1970s, and it allowed researchers to distinguish between chromosomes of similar sizes for the first time [16,17]. As conventional full karyotyping provides information on the number and gross appearance of chromosomes, it can be used to detect potentially pathogenic chromosomal aberrations including aneuploidy, deletions, duplications, and unbalanced translocations. Giemsa (G) banding has a highest resolution of 3–10 Mb [18].

A useful supplement to classical karyotyping is fluorescence *in situ* hybridisation (FISH). Fluorescent-tagged oligonucleotide probes complementary to a DNA sequence of interest are used to visualise chromosomal regions of interest. Locus-specific or gene-specific probes can be used to identify known aberrations that cause fetal or congenital abnormalities; for example, 22q11 deletions in DiGeorge syndrome, 7q11.23 deletions in Williams syndrome, and dystrophin mutations in Duchenne muscular dystrophy [19,20,21]. In another nice example of the clinical use of FISH, specific subtelomeric probes were used to identify an unbalanced subtelomeric translocation in a child with multiple congenital abnormalities, where classical cytogenetic analysis had indicated a normal karyotype [22]. Because FISH has to be “targeted” to a specific chromosomal anomaly, it requires a clinician to have a high degree of suspicion of that specific anomaly from the phenotype of the patient. Generally, fetal chromosome karyotyping, along with FISH where appropriate, is offered to families when a significant fetal anomaly is identified on ultrasound, or when there is a high risk of such an anomaly. In this population, karyotyping identifies a chromosomal anomaly in around 9% of cases [23].

### 2.3. Chromosomal Microarray (CMA)

Chromosomal Microarray, also known as array comparative genomic hybridisation (aCGH), detects CNVs that may be pathogenic, benign, or variants of unknown significance (VOUS). CMA has a higher resolution than G-band karyotyping, and can detect deletions or duplications as small as 1 kb, depending on the platform used [24]. However, these very high resolution arrays have disadvantages, including higher cost and increased detection of VOUS. Therefore, for clinical diagnostic purposes, microarrays with a resolution in the range of 10–400 kb are generally preferred [25,26]. The choice and resolution of the microarray “platform” used in prenatal diagnosis is the major discussion point occurring prior to CMA universal adoption for this role.

One limitation of CMA is that it is only able to detect unbalanced chromosomal rearrangements. Balanced rearrangements, such as reciprocal translocations, may lead to disease by disrupting genes, without detectable gains or losses at breakpoints [23,27]. However, in practice, many apparently balanced rearrangements detected by G-banding are not truly balanced at the DNA level, and CMA testing can be used to detect small regions of DNA loss or gain, and so clarify the exact nature of the rearrangement [28]. Many CMA platforms may not detect triploidy or low-level mosaicism [23,29]. However SNP based CMA platforms will. In the UK, quantitative fluorescence polymerase chain reaction (QF-PCR) is used prior to CMA as a rapid and cost-effective screen for common aneuploidy and triploidy [29].

Despite the limitations, CMA has been the diagnostic test of choice for several years in children and adults with developmental delay [30]. For fetuses with structural abnormalities, CMA has a diagnostic yield of ranging from 6% to 10% higher than chromosomal karyotyping [6,23,31]. Therefore, the UK is considering the introduction of prenatal CMA for all fetuses with structural anomalies only by the end of 2014 [32]. This is slightly different to the guidance from the American College of Obstetrics and Gynecology who recommend that CMA should be performed as the diagnostic procedure of choice on fetuses with a structural abnormality, and may also be performed in structurally normal fetuses undergoing invasive prenatal testing [33].

### 2.4. Non-Invasive Prenatal Testing

Up to 50% of cell-free DNA in the plasma of a pregnant woman is fetal-derived [34,35,36]. It consists of DNA fragments with a size range of 30–510 bps, and a median of 162 bps [37]. The cffDNA can be obtained non-invasively; therefore in recent years there has been huge interest in using it for prenatal genetic diagnosis. Non-invasive prenatal testing (NIPT) refers to sequencing cffDNA to identify genetic mutations in the fetus. NIPT is able to detect autosomal trisomies and sex chromosome aneuploidies [36,38,39,40,41], CNVs [42,43], identify the sex of the fetus [44], Rhesus status [45], and single gene disorders such as achondroplasia and cystic fibrosis [46,47,48].

Regarding clinical practice, in the United States and China, use of NIPT to detect aneuploidies and fetal sex is already widespread [49,50]. Implementation for single-gene disorders is much slower because of lower demand and higher technical challenges [48]. In the UK, NIPT is currently only being provided by the National Health Service for sex determination, genotyping of fetuses at risk of Rhesus disease, and some single-gene disorders. However, the “Reliable accurate prenatal non-invasive diagnosis” (RAPID) and “Non-invasive prenatal diagnosis for single gene disorders” (NIPSIGEN) studies are investigating how to expand the implementation, and UK health professionals generally view NIPT positively, therefore it is likely that provision will be expanded to other genomic disorders in the near future [51].

Two proof-of-concept studies published in 2012 showed that it is possible to sequence the whole genome of a fetus non-invasively using cffDNA, to a sufficient depth to be able to call SNVs, using parental haplotypes to distinguish fetal from maternal variants [52,53]. However, the sensitivity and specificity of the SNV calling are as yet insufficient to consider using this approach in clinical practice. The sensitivity and specificity of identification of *de novo* mutations were particularly low [53], and yet for diagnostics this is very important, as *de novo* mutations are especially likely to be pathogenic [9,54,55,56,57,58]. Therefore, to identify potentially pathogenic SNVs in fetuses with structural abnormalities, NGS on fetal DNA obtained through invasive methods remains, for now, the superior choice.

## 3. Prenatal Exome Sequencing

### 3.1. Exome Sequencing as both a Research Tool and a Diagnostic Test

NGS can be used to identify SNVs throughout the genome, thus it has a much higher resolution than cytogenetic approaches. Exome sequencing is often favoured over whole genome sequencing, as it targets only coding regions, which represent 1%–2% of the entire genome, but contain around 85% of the mutations that cause known genetic disorders [59,60,61].

The first report of exome sequencing as a method to discover the genetic cause of a Mendelian disease was made in 2010, with the identification of mutations in *DHODH* as the cause of Miller syndrome [62]. In the few short years since then, exome sequencing has proved to be a remarkably fruitful research tool. At least one hundred genes that harbour mutations causing Mendelian disease have been identified, and this rate of progress shows no signs of abating as yet [63]. Additionally, exome sequencing is increasingly being used in the clinical setting as a diagnostic test for patients with rare diseases. It has a diagnostic yield of around 25% [55,64].

### 3.2. Prenatal Exome Sequencing: Proof-of-Concept

Despite the success of NGS, only a handful of studies have used it for prenatal gene discovery or diagnosis. The first two such studies, both published in 2012, used NGS to identify aneuploidy and chromosomal rearrangements. Dan *et al*., used very low-coverage whole-genome sequencing to detect aneuploidies and unbalanced chromosome variants in 13/62 fetuses [65], and Talkowski *et al*., used whole genome “jumping library” sequencing of amniocytes to identify an apparently balanced *de novo* translocation that disrupts *CHD7*, causing CHARGE syndrome in a single fetus [66].

The next two studies used exome sequencing at a depth sufficient to identify SNVs, in a very small number of fetuses. Yang *et al*., performed exome sequencing on 250 patients with Mendelian disorders, four of which were fetuses from terminated pregnancies [55]. In one of the fetuses, which had Cornelia de Lange syndrome, they found the cause of disease, which was a *de novo* splicing variant in the known gene *NIPBL*. Finally, Filges *et al*., used exome sequencing to identify the cause of a recessive, lethal ciliopathy phenotype in one family [67]. They sequenced the parents, their unaffected daughter, and post-mortem samples from two fetuses that were affected by the disease, and found compound heterozygous mutations in *KIF14* in both affected fetuses.

### 3.3. Prenatal Exome Sequencing: Diagnostic Yield

In our recent study, we demonstrated that for fetuses with structural abnormalities, exome sequencing improves diagnostic yield over karyotyping and CMA alone [9]. The study was collaboration between the University of Birmingham (Birmingham, UK) and the Wellcome Trust Sanger Institute (Cambridge, UK), and it is the largest published cohort of fetuses with structural abnormalities to have been exome sequenced to date.

We gathered a cohort of 30 euploid fetuses with a diverse range of structural abnormalities detected by ultrasound. We exome sequenced them using the Illumina HiSeq platform in a trio design. That is, maternal and paternal samples were sequenced in addition to the affected fetus. We considered *de novo* rare coding mutations, and inherited recessive and X-linked rare coding mutations. It is important to distinguish between *de novo* and inherited mutations, in order to estimate the recurrence risk for future pregnancies. We considered SNVs and CNVs (which were identified directly from the exome sequencing reads using the CoNVex software [68]). These mutations were classified through a systematic manual process as highly likely to be causal, possibly causal, or unknown.

A list of 77 potential candidate *de novo* coding or splicing variants (mean = 2.6 per fetus, range 0–5) was found. Thirty-four (mean = 1.13 per fetus, range 0–4) variants were then confirmed as truly *de novo* as a result of subsequent capillary sequencing, in-keeping with the known germline mutation rate [69,70]. For 3/30 fetuses we identified mutations that are highly likely to be causal [9]. That is, similar mutations in the same gene have previously been shown to cause a very similar phenotype in humans. One of these mutations was in the *FGFR3* gene which is a negative regulator of bone growth; the features of the fetus were that of a lethal skeletal dysplasia thus supporting that the genetic abnormality detected on exome sequencing was pathogenic. Another mutation was found in a different fetus in *COL2A1*, mutations in this gene are known to cause type II collagenopathies including heart and limb defects, although the particular mutation that we detected had not previously been reported. This second fetus had increased nuchal translucency, tricuspid regurgitation, abnormal hands and feet and bilateral talipes, again supporting the results of exome sequencing. A third fetus which had ventriculomegaly and agenesis of the corpus collosum demonstrated a *de novo* Xp22.2 deletion which resulted in the removal of most of the *OFD1* gene which is known to cause orofaciodigital syndrome, and is associated with absence of the corpus callosum is approximately 40% [71].

The diagnostic yield of this study was 10%. Only one of these mutations was a CNV detected by CMA, highlighting the utility of exome sequencing, and increasing the detection rate of prenatal genetic abnormalities over that achieved by karyotyping and CMA alone [6,23,31].

Nevertheless, our diagnostic rate is lower than that found in exome sequencing studies of rare postnatal diseases [55,64]. There are several possible reasons for this. Our estimate of 10%, being based on a relatively small sample size, has a broad confidence interval. Furthermore, it is likely that in some cases, mutations in the same gene will have different phenotypic manifestations between prenatal and postnatal stages of development [72]. Also, in some cases we only had phenotypic data based on ultrasound scans. There are many phenotypes that cannot be identified from an ultrasound scan. Interpreting the clinical significance of the many unknown variants identified is generally the most challenging aspect of any exome sequencing study of rare disease [73,74]. For the reasons described, doing so in the context of prenatal phenotypic data is particularly difficult. However, as more exome sequencing is performed, databases of pathogenic mutations such as the DECIPHER database will be expanded [75], the diagnostic yield of exome sequencing, including in the prenatal context, is likely to increase. The ability to match SNVs to organ-specific anomalies, for example, the heart, would be particularly useful and may allow a “targeted” approach.

## 4. The Ethics of Prenatal Exome Sequencing as a Screening Tool

There is no technical reason why exome sequencing of fetal DNA obtained during pregnancy could not be used on structurally normal fetuses, as a screening tool rather than a diagnostic test. While this is not a proposal that is likely to be implemented in the near future, it is a responsibility of the prenatal genetic diagnostics community to begin to consider the relevant ethical issues [76].

The many thorny ethical issues surrounding exome sequencing in the clinical postnatal context have been extensively debated, chief among which are whether to report incidental findings, and how to report VOUS. Last year, the American College of Medical Genetics and Genomics (ACMG) recommended that mutations in a set of 57 known disease genes should always be reported where sequencing is used as a clinical test [77]. Fetuses were excluded from the ACMG recommendations. They have since revised these recommendations, saying patients have a right to opt out of receiving information on incidental findings [78]. The European Society of Human Genetics have adopted rather more conservative guidelines preferring a targeted approach to avoid discovering incidental findings [79], and the Deciphering Developmental Disorders project which is investigating the causes of rare genetic diseases in children in the UK is not returning incidental findings to participants under any circumstances [80]. Some researchers and ethicists object to the ACMG recommendations on the grounds that the line between “actionable” and “non-actionable” conditions is far from clear, and the probabilistic nature of the link between genotype and phenotype will result in unnecessary distress for a proportion of families who receive information about mutations which will in fact never cause them to develop disease [81]. Some have argued that routinely returning incidental findings regarding a list of genes amounts to genomic screening [82].

When considering prenatal sequencing as a screening tool, the ethical dilemmas are similar but amplified, partly due to the possibility of termination of the pregnancy. If prenatal sequencing were to be used as a screening tool, in some cases, it would identify a variant that causes a severe, distressing, and lethal phenotype and is highly penetrant, at an earlier stage than an ultrasound scan could have found structural abnormalities. An example of such a variant might be missense changes in *FGFR3* that cause thanataphoric dysplasia [8,9]. In this scenario, earlier detection is undoubtedly better for families. It avoids potentially devastating news later in pregnancy, in the neonatal period, or even later in childhood when developmental delay is clinically apparent. If the families elect to terminate the pregnancy, distress is generally less severe at an early stage of pregnancy [83]. For families who choose to continue with the pregnancy, early diagnosis may offer a more accurate prognosis, more time to prepare, and in some cases the option to start treatments earlier. Therefore, these families would definitely benefit from prenatal sequencing.

In other, less clear-cut cases, the disadvantages of prenatal sequencing as a screening tool may outweigh the advantages. Parents are potentially at risk of “truth dumping”, whereby a large quantity of information is given to parents and they are expected to interpret the information which professionals are unable to interpret with certainty, and then make a decision. Identification of “normal” genomic variation and VOUS is virtually inevitable during prenatal sequencing. For example, a predicted pathogenic variant may be identified in a known developmental disorder gene, but if it has never been reported before it may be very difficult to accurately predict the phenotype. The ethical issues of returning VOUS to families have been considered in the context of CNVs discovered by CMA. Some research suggests that receiving information on VOUS during pregnancy can be very stressful and distressing [84]. Therefore, some researchers and clinicians think that they should not be reported to families, and that their detection should be limited in the first place by using targeted tests [26,85]. Others think that it is paternalistic to withhold this information [86]. If VOUS were to be returned, it is imperative that families receive extensive genetic counselling pre- and post-test [33]. These issues are still under debate, but it would be important for clinicians and researchers to come to a consensus on the issue of reporting VOUS, prior to any use of prenatal exome sequencing as a screening technique, because interpreting mutations identified by exome sequencing is generally more difficult than those identified by CMA, and there will be a higher number of VOUS identified.

Another question is whether to return to families information on mutations that are likely to cause late-onset disease, and/or have incomplete penetrance, such as a *BRCA1* variant that confers an 80% risk of developing cancer later in life [87]. Some argue that families have a right to this information to do with what they will, even if it will result in increased termination rates, and termination of some healthy fetuses [76]. An alternative is to do more targeted sequencing based on the indication for the test, so as to avoid incidental diagnoses.

Additional problems include issues surrounding paternity, both in terms of needing correct information to aid the diagnosis of *de novo* mutations, but also decision making when couples disagree. It also reignites the perpetual ethical debate regarding the rights of the fetus itself. The potential to use exome sequencing to test for non-medical genetic markers and consequently create a “designer baby” is also a fear.

There are currently more questions than answers regarding the ethics of prenatal exome sequencing as a screening tool. Nevertheless, many pertinent issues have already been thoroughly discussed in the context of postnatal clinical sequencing, or interpretation of prenatal CMA results. While prenatal exome sequencing clearly poses additional specific ethical challenges, it is likely that with continued open debate amongst clinicians and researchers, along with sensitive and thorough genetic counselling to families, these can be overcome.

## 5. Next-Generation Sequencing: The Future of Prenatal Genetic Diagnostics

From a scientific perspective, it seems likely that next-generation sequencing is the future of prenatal genetic diagnostics. Nevertheless, many questions remain to be answered before prenatal next-generation sequencing could become widespread. These include issues of cost effectiveness, clinical utility, ethics, and interpretation of mutations. To address some of these, the Wellcome Trust and the Department of Health in the UK have awarded a Health Innovation Challenge Fund grant to the collaborative Prenatal Assessment of Genomes and Exomes (PAGE) project. This will involve the Wellcome Trust Sanger Institute, the University of Cambridge, the University of Birmingham, Birmingham Women’s Foundation Trust, University College London and Great Ormond Street Hospital (London, UK). One thousand fetuses with structural abnormalities, along with maternal and paternal samples, will undergo exome sequencing or whole genome sequencing. Qualitative work and an ethical review will also be performed to address the many issues this rapidly developing area is raising. The results of this study are expected to yield insights into the genetic causes of fetal abnormalities, and pave the way scientifically, clinically and socially for large-scale implementation of NGS in the UK’s prenatal arena.

In this review we have focused on exome sequencing, as it is currently more cost-efficient than whole-genome sequencing for clinical diagnostic purposes. However, for several reasons, we predict an eventual move towards whole-genome sequencing rather than exome sequencing for clinical diagnostic purposes, including in prenatal samples. First, there are many examples of non-coding mutations that can cause congenital abnormalities including pancreatic agenesis and malformations of the digits [88,89,90,91]. These variants would usually not be detected by exome sequencing. Additionally, while the costs of NGS are falling rapidly [92], if the costs of the exome capture step do not fall in line with this, at some point whole-genome sequencing may become more cost-effective than exome sequencing [93]. Finally, a major reason why whole-genome sequencing is currently often avoided is that interpretation of non-coding variants is very difficult. However, with large-scale whole-genome projects being planned, this is also likely to start becoming easier [94].

## 6. Conclusions

Next Generation Sequencing and examination of exomes is a powerful tool. In fetuses with congenital malformations it is helpful when delineating prenatal prognosis to be able to exclude chromosomal anomalies. However, the use of these new genetic techniques potentially allows identification of causative single gene anomalies and equally may allow the exclusion of such risk and the reassurance to parents that the underlying aetiology of fetal structural anomalies is “*de-novo*”.

The PAGE study, undertaken in the UK from 2014 to 2016 will allow us to collect information on the use of such technology in prenatal diagnosis and to evaluate the potential for its use in clinical practice.

## References

[1] Springett A., Morris J.K. (2010). Congenital Anomaly Statistics, England and Wales.

[2] Vlastos I.M., Koudoumnakis E., Houlakis M., Nasika M., Griva M., Stylogianni E. (2009). Cleft lip and palate treatment of 530 children over a decade in a single centre. Int. J. Pediatr. Otorhinolaryngol..

[3] Schmidt W., Schroeder T.M., Buchinger G., Kubli F. (1982). Genetics, pathoanatomy and prenatal diagnosis of Potter I syndrome and other urogenital tract diseases. Clin. Genet..

[4] Verity C., Firth H., French-Constant C. (2003). Congenital abnormalities of the central nervous system. J. Neurol. Neurosurg. Psychiatry.

[5] Cereda A., Carey J.C. (2012). The trisomy 18 syndrome. Orphanet J. Rare Dis..

[6] Hillman S.C., McMullan D.J., Hall G., Togneri F.S., James N., Maher E.J., Meller C.H., Williams D., Wapner R.J., Maher E.R. (2013). Use of prenatal chromosomal microarray: Prospective cohort study and systematic review and meta-analysis. Ultrasound Obstet. Gynecol..

[7] Skinner M.A., Safford S.D., Reeves J.G., Jackson M.E., Freemerman A.J. (2008). Renal aplasia in humans is associated with RET mutations. Am. J. Hum. Genet..

[8] Rousseau F., El Ghouzzi V., Delezoide A.L., Legeai-Mallet L., le Merrer M., Munnich A., Bonaventure J. (1996). Missense *FGFR3* mutations create cysteine residues in thanatophoric dwarfism type I (TD1). Hum. Mol. Genet..

[9] Carss K.J., Hillman S.C., Parthiban V., McMullan D.J., Maher E.R., Kilby M.D., Hurles M.E. (2014). Exome sequencing improves genetic diagnosis of structural fetal abnormalities revealed by ultrasound. Hum. Mol. Genet..

[10] Firth H.V., Wright C.F. (2011). The Deciphering Developmental Disorders (DDD) study. Dev. Med. Child Neurol..

[11] Tabor A., Philip J., Madsen M., Bang J., Obel E.B., Norgaard-Pedersen B. (1986). Randomised controlled trial of genetic amniocentesis in 4606 low-risk women. Lancet.

[12] Smidt-Jensen S., Permin M., Philip J., Lundsteen C., Zachary J.M., Fowler S.E., Grünig K. (1992). Randomised comparison of amniocentesis and transabdominal and transcervical chorionic villus sampling. Lancet.

[13] Group CCC-ACT (1989). Multicentre randomised clinical trial of chorion villus sampling and amniocentesis. Lancet.

[14] Tabor A., Vestergaard C.H., Lidegaard O. (2009). Fetal loss rate after chorionic villus sampling and amniocentesis: An 11-year national registry study. Ultrasound Obstet. Gynecol..

[15] Lo Y.M., Corbetta N., Chamberlain P.F., Rai V., Sargent I.L., Redman C.W., Wainscoat J.S. (1997). Presence of fetal DNA in maternal plasma and serum. Lancet.

[16] Caspersson T., Farber S., Foley G.E., Kudynowski J., Modest E.J., Simonsson E., Waqh U., Zech L. (1968). Chemical differentiation along metaphase chromosomes. Exp. Cell Res..

[17] Sumner A.T., Evans H.J., Buckland RA. (1971). New technique for distinguishing between human chromosomes. Nat. New Biol..

[18] Shaffer L.G., Bejjani B.A. (2004). A cytogeneticist’s perspective on genomic microarrays. Hum. Reprod. Update.

[19] Driscoll D.A., Salvin J., Sellinger B., Budarf M.L., McDonald-McGinn D.M., Zackai E.H., Emanuel B.S. (1993). Prevalence of 22q11 microdeletions in DiGeorge and velocardiofacial syndromes: Implications for genetic counselling and prenatal diagnosis. J. Med. Genet..

[20] Nickerson E., Greenberg F., Keating M.T., McCaskill C., Shaffer L.G. (1995). Deletions of the elastin gene at 7q11.23 occur in approximately 90% of patients with Williams syndrome. Am. J. Hum. Genet..

[21] Ligon A.H., Kashork C.D., Richards C.S., Shaffer L.G. (2000). Identification of female carriers for Duchenne and Becker muscular dystrophies using a FISH-based approach. Eur. J. Hum. Genet..

[22] Brackley K.J., Kilby M.D., Morton J., Whittle M.J., Knight S.J., Flint J. (1999). A case of recurrent congenital fetal anomalies associated with a familial subtelomeric translocation. Prenat. Diagn..

[23] Wapner R.J., Martin C.L., Levy B., Ballif B.C., Eng C.M., Zachary J.M., Melissa Savage M.S., Lawrence D., Platt M.D., Daniel Saltzman M.D. (2012). Chromosomal microarray versus karyotyping for prenatal diagnosis. N. Engl. J. Med..

[24] Hillman S.C., Pretlove S., Coomarasamy A., McMullan D.J., Davison E.V., Maher E.R., Kilby M.D. (2011). Additional information from array comparative genomic hybridization technology over conventional karyotyping in prenatal diagnosis: A systematic review and meta-analysis. Ultrasound Obstet. Gynecol..

[25] Shaffer L.G., Dabell M.P., Rosenfeld J.A., Neill N.J., Ballif B.C., Coppinger J., Diwan N.R., Chong K., Shohat M., Chitayat D. (2012). Referral patterns for microarray testing in prenatal diagnosis. Prenat. Diagn..

[26] Vanakker O., Vilain C., Janssens K., Van der Aa N., Smits G., Bandelier C., Blaumeiser B., Bulk S., Caberq J.H., de Leener A. (2014). Implementation of genomic arrays in prenatal diagnosis: The Belgian approach to meet the challenges. Eur. J. Med. Genet..

[27] Bodrug S.E., Roberson J.R., Weiss L., Ray P.N., Worton R.G., van Dyke D.L. (1990). Prenatal identification of a girl with a t(X;4)(p21;q35) translocation: Molecular characterisation, paternal origin, and association with muscular dystrophy. J. Med. Genet..

[28] De Gregori M., Ciccone R., Magini P., Pramparo T., Gimelli S., Messa J., Novara F., Vetro A., Rossi E., Maraschio P. (2007). Cryptic deletions are a common finding in “balanced” reciprocal and complex chromosome rearrangements: A study of 59 patients. J. Med. Genet..

[29] Hillman S.C., McMullan D.J., Maher E.R., Kilby M.D. (2013). The use of chromosomal microarray in prenatal diagnosis. Obstet. Gynaecol..

[30] UKGTN (2009). UK Genetic Testing Network arrayCGH Commissioning Workshop.

[31] Valduga M., Philippe C., Bach Segura P., Thiebaugeorges O., Miton A., Beri M., Bonnet C., Nemos C., Foliquet B., Jonveaux P. (2010). A retrospective study by oligonucleotide array-CGH analysis in 50 fetuses with multiple malformations. Prenat. Diagn..

[32] Clinical Genetics Clinical Reference Group (2014). Personal communication.

[33] The American College of Obstetricians and Gynecologists Committee on Genetics Society for Maternal-Fetal Medicine (2013). Committee Opinion No. 581: The use of chromosomal microarray analysis in prenatal diagnosis. Obstet. Gynecol..

[34] Lo Y.M., Tein M.S., Lau T.K., Haines C.J., Leung T.N., Poon P.M., Wainscoat J.S., Johnson P.J., Chang A.M., Hjelm N.M. (1998). Quantitative analysis of fetal DNA in maternal plasma and serum: Implications for noninvasive prenatal diagnosis. Am. J. Hum. Genet..

[35] Lun F.M., Chiu R.W., Allen Chan K.C., Yeung Leung T., Kin Lau T., Dennis Lo Y.M. (2008). Microfluidics digital PCR reveals a higher than expected fraction of fetal DNA in maternal plasma. Clin. Chem..

[36] Palomaki G.E., Deciu C., Kloza E.M., Lambert-Messerlian G.M., Haddow J.E., Neveux L.M., Ehrich M., van den Boom D., Bombard A.T., Grody W.W. (2012). DNA sequencing of maternal plasma reliably identifies trisomy 18 and trisomy 13 as well as Down syndrome: An international collaborative study. Genet. Med..

[37] Fan H.C., Blumenfeld Y.J., Chitkara U., Hudgins L., Quake S.R. (2010). Analysis of the size distributions of fetal and maternal cell-free DNA by paired-end sequencing. Clin. Chem..

[38] Nicolaides K.H., Syngelaki A., Gil M., Atanasova V., Markova D. (2013). Validation of targeted sequencing of single-nucleotide polymorphisms for non-invasive prenatal detection of aneuploidy of chromosomes 13, 18, 21, X, and Y. Prenat. Diagn..

[39] Samango-Sprouse C., Banjevic M., Ryan A., Sigurjonsson S., Zimmermann B., Hill M., Hall M.P., Westemeyer M., Saucier J., Demko Z. (2013). SNP-based non-invasive prenatal testing detects sex chromosome aneuploidies with high accuracy. Prenat. Diagn..

[40] Hooks J., Wolfberg A.J., Wang E.T., Struble C.A., Zahn J., Juneau K., Mohseni M., Huang S., Bogard P., Song K. (2014). Non-invasive risk assessment of fetal sex chromosome aneuploidy through directed analysis and incorporation of fetal fraction. Prenat. Diagn..

[41] Jiang F., Ren J., Chen F., Zhou Y., Xie J., Dan S., Su Y., Xie J.H., Yin B.M., Su W. (2012). Noninvasive Fetal Trisomy (NIFTY) test: An advanced noninvasive prenatal diagnosis methodology for fetal autosomal and sex chromosomal aneuploidies. BMC Med. Genomics.

[42] Lau T.K., Jiang F.M., Stevenson R.J., Lo T.K., Chan L.W., Chan M.K., Lo P.S., Wang W., Zhang H.Y., Chen F. (2013). Secondary findings from non-invasive prenatal testing for common fetal aneuploidies by whole genome sequencing as a clinical service. Prenat. Diagn..

[43] Ge H., Huang X., Li X., Chen S., Zheng J., Jiang H., Zhang C., Pan X., Chen F., Chen N. (2013). Noninvasive prenatal detection for pathogenic CNVs: The application in alpha-thalassemia. PLoS One.

[44] Wright C.F., Wei Y., Higgins J.P., Sagoo G.S. (2012). Non-invasive prenatal diagnostic test accuracy for fetal sex using cell-free DNA a review and meta-analysis. BMC Res. Notes.

[45] Moise K.J., Boring N.H., O’Shaughnessy R., Simpson L.L., Wolfe H.M., Baxter J.K., Polzin W., Eddleman K.A., Hassan S.S., Skupski D. (2013). Circulating cell-free fetal DNA for the detection of RHD status and sex using reflex fetal identifiers. Prenat. Diagn..

[46] Lim J.H., Kim M.J., Kim S.Y., Kim H.O., Song M.J., Kim M.H., Park S.Y., Yang J.H., Ryu H.M. (2011). Non-invasive prenatal detection of achondroplasia using circulating fetal DNA in maternal plasma. J. Assist. Reprod. Genet..

[47] Bustamante-Aragones A., Gallego-Merlo J., Trujillo-Tiebas M.J., de Alba M.R., Gonzalez-Gonzalez C., Glover G., Diego-Alvarez D., Ayuso C., Ramos C. (2008). New strategy for the prenatal detection/exclusion of paternal cystic fibrosis mutations in maternal plasma. J. Cyst. Fibros..

[48] Lench N., Barrett A., Fielding S., McKay F., Hill M., Jenkins L., White H., Chitty L.S. (2013). The clinical implementation of non-invasive prenatal diagnosis for single-gene disorders: Challenges and progress made. Prenat. Diagn..

[49] Agarwal A., Sayres L.C., Cho M.K., Cook-Deegan R., Chandrasekharan S. (2013). Commercial landscape of noninvasive prenatal testing in the United States. Prenat. Diagn..

[50] Song Y., Liu C., Qi H., Zhang Y., Bian X., Liu J. (2013). Noninvasive prenatal testing of fetal aneuploidies by massively parallel sequencing in a prospective Chinese population. Prenat. Diagn..

[51] Hill M., Karunaratna M., Lewis C., Forya F., Chitty L. (2013). Views and preferences for the implementation of non-invasive prenatal diagnosis for single gene disorders from health professionals in the United Kingdom. Am. J. Med. Genet. Part A.

[52] Fan H.C., Gu W., Wang J., Blumenfeld Y.J., El-Sayed Y.Y., Quake S.R. (2012). Non-invasive prenatal measurement of the fetal genome. Nature.

[53] Kitzman J.O., Snyder M.W., Ventura M., Lewis A.P., Qiu R., Simmons L.E., Gammill H.S., Rubens C.E., Santillan D.A., Murray J.C. (2012). Noninvasive whole-genome sequencing of a human fetus. Sci. Transl. Med..

[54] Veltman J.A., Brunner H.G. (2012). *De novo* mutations in human genetic disease. Nat. Rev. Genet..

[55] Yang Y., Muzny D.M., Reid J.G., Bainbridge M.N., Willis A., Ward P.A., Braxton A., Beuten J., Xia F., Niu Z. (2013). Clinical whole-exome sequencing for the diagnosis of mendelian disorders. N. Engl. J. Med..

[56] O’Roak B.J., Deriziotis P., Lee C., Vives L., Schwartz J.J., Girirajan S., Karakoc E., MacKenzie A.P., Ng S.B., Baker C.B. (2011). Exome sequencing in sporadic autism spectrum disorders identifies severe *de novo* mutations. Nat. Genet..

[57] De Ligt J., Willemsen M.H., van Bon B.W., Kleefstra T., Yntema H.G., Kroes T., Anneke T., Vulto-van Silfhout M.D., David A., Koolen M.D. (2012). Diagnostic exome sequencing in persons with severe intellectual disability. N. Engl. J. Med..

[58] Zaidi S., Choi M., Wakimoto H., Ma L., Jiang J., Overton J.D., Romano-Adesman A., Bjornson R.D., Breitbart R.E., Brown K.K. (2013). De novo mutations in histone-modifying genes in congenital heart disease. Nature.

[59] Ng S.B., Turner E.H., Robertson P.D., Flygare S.D., Bigham A.W., Lee C., Shaffer T., Wong M., Bhattacharjee A., Eichler E.E. (2009). Targeted capture and massively parallel sequencing of 12 human exomes. Nature.

[60] Bainbridge M.N., Wang M., Burgess D.L., Kovar C., Rodesch M.J., D’Ascenzo M., Kitzman J., Wu Y.Q., Newsham I., Richmond T.A. (2010). Whole exome capture in solution with 3 Gbp of data. Genome Biol..

[61] Stenson P.D., Ball E.V., Howells K., Phillips A.D., Mort M., Cooper D.N. (2009). The Human Gene Mutation Database: Providing a comprehensive central mutation database for molecular diagnostics and personalized genomics. Hum. Genomics.

[62] Ng S.B., Buckingham K.J., Lee C., Bigham A.W., Tabor H.K., Dent K.M., Huff C.D., Shannon P.T., Jabs E.W., Nickerson D.A. (2010). Exome sequencing identifies the cause of a mendelian disorder. Nat. Genet..

[63] Wang Z., Liu X., Yang B.Z., Gelernter J. (2013). The role and challenges of exome sequencing in studies of human diseases. Front. Genet..

[64] Gahl W.A., Markello T.C., Toro C., Fajardo K.F., Sincan M., Gill F., Carlson-Donohoe H., Gropman A., Pierson T.M., Golas G. (2012). The National Institutes of Health Undiagnosed Diseases Program: Insights into rare diseases. Genet. Med..

[65] Dan S., Chen F., Choy K.W., Jiang F., Lin J., Xuan Z., Wang W., Chen S., Li X., Jiang H. (2012). Prenatal detection of aneuploidy and imbalanced chromosomal arrangements by massively parallel sequencing. PLoS One.

[66] Talkowski M.E., Ordulu Z., Pillalamarri V., Benson C.B., Blumenthal I., Connolly S., Hanscom C., Hussain N., Pereira S., Picker J. (2012). Clinical diagnosis by whole-genome sequencing of a prenatal sample. N. Engl. J. Med..

[67] Filges I., Nosova E., Bruder E., Tercanli S., Townsend K., Gibson W., Röthlisberger B., Heinimann K., Hall J., Gregory-Evans C. (2013). Exome sequencing identifies mutations in KIF14 as a novel cause of an autosomal recessive lethal fetal ciliopathy phenotype. Clin. Genet..

[68] CoNVex: Copy number variants from exomes. Availableonline:ftp://ftp.sanger.ac.uk/pub/users/pv1/CoNVex/.

[69] Rauch A., Wieczorek D., Graf E., Wieland T., Endele S., Schwarzmayr T. (2012). Range of genetic mutations associated with severe non-syndromic sporadic intellectual disability: An exome sequencing study. Lancet.

[70] Awadalla P., Gauthier J., Myers R.A., Casals F., Hamdan F.F., Griffing A.R., Côté M., Henrion E., Spiegelman D., Tarabeux J. (2010). Direct measure of the de novo mutation rate in autism and schizophrenia cohorts. Am. J. Hum. Genet..

[71] Bischoff I.J., Zeschnigk C., Horn D., Wellek B., Riess A., Wessels M., Willems P., Jensen P., Busche A., Bekkebraten J. (2013). Novel mutations including deletions of the entire OFD1 gene in 30 families with type 1 orofaciogdigital syndrome: A study of the extensive clinical variability. Hum. Mutat..

[72] Mefford H.C., Clauin S., Sharp A.J., Moller R.S., Ullmann R., Kapur R., Pinkel D., Cooper G.M., Ventura M., Ropers H.H. (2007). Recurrent reciprocal genomic rearrangements of 17q12 are associated with renal disease, diabetes, and epilepsy. Am. J. Hum. Genet..

[73] Stanley C.M., Sunyaev S.R., Greenblatt M.S., Oetting W.S. (2014). Clinically relevant variants—Identifying, collecting, interpreting, and disseminating: The 2013 annual scientific meeting of the human genome variation society. Hum. Mutat..

[74] Isakov O., Perrone M., Shomron N. (2013). Exome sequencing analysis: A guide to disease variant detection. Methods Mol. Biol..

[75] Bragin E., Chatzimichali E.A., Wright C.F., Hurles M.E., Firth H.V., Bevan A.P., Swaminathan G.J. (2014). Decipher: Database for the interpretation of phenotype-linked plausibly pathogenic sequence and copy-number variation. Nucl. Acid. Res..

[76] Yurkiewicz I.R., Korf B.R., Lehmann L.S. (2014). Prenatal whole-genome sequencing—Is the quest to know a fetus’s future ethical?. N. Engl. J. Med..

[77] Green R.C., Berg J.S., Grody W.W., Kalia S.S., Korf B.R., Martin C.L., Amy McGuire J.D., Nussbaum R.L., O’Daniel J.M., Ormond K.E. (2013). ACMG recommendations for reporting of incidental findings in clinical exome and genome sequencing. Genet. Med..

[78] American College of Medical Genetics and Genomics ACMG Updates Recommendation on “Opt Out” for Genome Sequencing Return of Results 2014. Availableonline:https://www.acmg.net/docs/Release_ACMGUpdatesRecommendations_final.pdf.

[79] Van El C.G., Cornel M.C., Borry P., Hastings R.J., Fellmann F., Hodgson S.V., Howard H.C., Cambon-Thomsen A., Knoppers B.M., Meijers-Heijboer H. (2013). Whole-genome sequencing in health care. Recommendations of the European Society of Human Genetics. Eur. J. Hum. Genet..

[80] Middleton A., Parker M., Wright C.F., Bragin E., Hurles M.E., Study D.D.D. (2013). Empirical research on the ethics of genomic research. Am. J. Med. Genet. Part A.

[81] Holtzman N.A. (2013). ACMG recommendations on incidental findings are flawed scientifically and ethically. Genet. Med..

[82] Wright C.F., Middleton A., Burton H., Cunningham F., Humphries S.E., Hurst J., Birney E., Firth H. (2013). Policy challenges of clinical genome sequencing. BMJ.

[83] Hern W.M. (2014). Fetal diagnostic indications for second and third trimester outpatient pregnancy termination. Prenat. Diagn..

[84] Bernhardt B.A., Soucier D., Hanson K., Savage M.S., Jackson L., Wapner R.J. (2013). Women’s experiences receiving abnormal prenatal chromosomal microarray testing results. Genet. Med..

[85] De Jong A., Dondorp W.J., Macville M.V., de Die-Smulders C.E., van Lith J.M., de Wert G.M. (2014). Microarrays as a diagnostic tool in prenatal screening strategies: Ethical reflection. Hum. Genet..

[86] McGillivray G., Rosenfeld J.A., McKinlay Gardner R.J., Gillam L.H. (2012). Genetic counselling and ethical issues with chromosome microarray analysis in prenatal testing. Prenat. Diagn..

[87] Ford D., Easton D.F., Stratton M., Narod S., Goldgar D., Devilee P., Bishop D.T., Weber B., Lenoir G., Chang-Claude J. (1998). Genetic heterogeneity and penetrance analysis of the *BRCA1* and *BRCA2* genes in breast cancer families. The Breast Cancer Linkage Consortium. Am. J. Hum. Genet..

[88] Weedon M.N., Cebola I., Patch A.M., Flanagan S.E., de Franco E., Caswell R., Rodríguez-Seguí S.A., Shaw-Smith C., Cho C.H.H., Allen H.L. (2014). Recessive mutations in a distal *PTF1A* enhancer cause isolated pancreatic agenesis. Nat. Genet..

[89] Benko S., Gordon C.T., Mallet D., Sreenivasan R., Thauvin-Robinet C., Brendehaug A., Thomas S., Bruland O., David M., Nicolino M. (2011). Disruption of a long distance regulatory region upstream of *SOX9* in isolated disorders of sex development. J. Med. Genet..

[90] Dathe K., Kjaer K.W., Brehm A., Meinecke P., Nurnberg P., Neto J.C., Brunoni D., Tommerup N., Ott C.E., Klopocki E. (2009). Duplications involving a conserved regulatory element downstream of *BMP2* are associated with brachydactyly type A2. Am. J. Hum. Genet..

[91] Lettice L.A., Heaney S.J., Purdie L.A., Li L., de Beer P., Oostra B.A., Goode D., Elgar G., Hill R.E., de Graaff E. (2003). A long-range Shh enhancer regulates expression in the developing limb and fin and is associated with preaxial polydactyly. Hum. Mol. Genet..

[92] Wetterstrand K.A. DNA sequencing costs: Data from the NHGRI Genome Sequencing Program (GSP). National Human Genome Research Institute 2013. Availableonline:https://www.genome.gov/sequencingcosts.

[93] Biesecker L.G., Shianna K.V., Mullikin J.C. (2011). Exome sequencing: The expert view. Gen. Biol..

[94] Prime Minister’s Office Press release: DNA tests to revolutionise fight against cancer and help 100,000 NHS patients 2012. Availableonline:https://www.gov.uk/government/news/dna-tests-to-revolutionise-fight-against-cancer-and-help-100000-nhs-patients.

